# Impact of campus living conditions on Chinese medical school students’ mental health during the COVID-19 campus lockdown: the chain mediating role of cognitive reappraisal and expression suppression

**DOI:** 10.3389/fpsyt.2023.1171425

**Published:** 2023-05-17

**Authors:** Wei Zhang, Long Huang, Fengyun Xu, Hairong Liu, Guoping Wang

**Affiliations:** ^1^School of Marxism, Wannan Medical College, Wuhu, China; ^2^School of Humanities and Management, Wannan Medical College, Wuhu, China; ^3^Anhui Key Laboratory of Philosophy and Social Sciences for Public Health Crisis Management, Wuhu, China

**Keywords:** COVID-19 pandemic, campus lockdown, medical school students, emotion regulation strategies, mental health

## Abstract

**Objective:**

To investigate the effect of changes in campus living conditions related to the Corona Virus Disease 2019 (COVID-19) pandemic on medical school students’ mental health status, to explore the mediating role of emotion regulation strategies, and to provide effective suggestions for promoting medical school students’ mental health.

**Methods:**

A self-report questionnaire, an emotion regulation questionnaire (ERQ), and psychological questionnaires for emergent events of public health (PQEEPH) were used to interview 998 medical school students who experienced campus lockdowns during the COVID-19 pandemic.

**Results:**

The mean total PQEEPH score was 3.66 ± 3.06. The degrees of inconvenience in daily life and change in routine and expression suppression as an emotion regulation strategy were significantly positively correlated with all PQEEPH dimensions. Cognitive reappraisal was significantly negatively associated with depression, neurosis, obsessive–compulsive anxiety, and hypochondriasis (*ps* < 0.05). Cognitive reappraisal and expression suppression demonstrated a chain mediating role between the degree of inconvenience in life and mental health and between the degree of change in routine and mental health (*F* = 32.883, 41.051, *ps* < 0.05).

**Conclusion:**

Campus lockdown management significantly impacts medical school students’ mental health. Extensive use of cognitive reappraisal and expression suppression can reduce students’ adverse psychological reactions during campus lockdowns to an extent.

## 1. Introduction

Since 2020, the novel SARS-CoV-2 has caused a rapidly expanding, life-threatening pandemic (the COVID-19 pandemic) (1). Most schools worldwide were closed and students had to complete courses delivered entirely remotely to prevent the spread of the pandemic in the spring semester of 2020 (2). Most students have returned to school attending classes (either online or offline) following necessary public health precautions since the fall semester of 2020. These precautions have been called the “new normal” in the US and “regular measures implemented to control epidemic” in China (3, 4). Because of the continuing mutation, shorter incubation period, and higher rates of asymptomatic infection, the pandemic situation became more pressing in the first half of 2022, and some colleges and universities in China had to adopt a 1–3-month campus lockdown policy (or “static management”) in response to local COVID-19 outbreaks (5). Accordingly, to cope with this situation, different degrees of campus lockdown measures were adopted to set clear boundaries between areas on and off campus and separate internal and external personnel (6). Furthermore, colleges and universities had implemented various degrees of pandemic prevention and control measures, including strict access management; strict student, faculty, and staff management; online study and examinations; dine-in option banishment; closure of public places on campus; campus-wide nucleic acid amplification tests; and dormitory quarantine with necessities delivered by volunteers (7–11).

Research has found that the length of closure, isolated living conditions, health habits, physical exercise, and sleep conditions had an important impact on people’s mental health during the pandemic lockdown, and that occupation, age, gender, and cultural background were also significant factors (12–16). However, campus lockdown management was not handled in the same way as general social lockdown management. The aforementioned studies placed more emphasis on the evaluation of objective factors in the lockdown, while the quantification of campus life and management factors was inadequate. As research on the impact of these subjective experiences on mental health is not sufficient, it is difficult to propose effective response measures and improvement suggestions for campus lockdown management.

The pandemic and prolonged campus lockdown have changed the campus routine, reduced the convenience of campus life, and adversely affected college students’ work and leisure (17), resulting in social isolation, loneliness, and relative deprivation (18, 19). Furthermore, these factors may have caused psychological stress, leading to negative emotions such as depression, neurosis, obsessive–compulsive anxiety, fear, and hypochondriasis (20–23). This means that the greater the inconveniences to one’s life and the higher the change in routine, the stronger the psychological reaction and the lower the level of psychological health. Consequently, we proposed the following hypothesis:

*Hypothesis 1*: Campus living conditions would positively predict medical school students’ psychological responses.

Emotion regulation is the management of people’s emotions, including when and how they express and experience them (24). Cognitive reappraisal is an adaptive emotion regulation strategy that helps to enhance the positive emotional experience of individuals and weaken their negative emotional experience. Meanwhile, expression suppression is a non-adaptive emotion regulation strategy and is more likely to induce various psychological disorders. The appropriate use of emotion regulation strategies can effectively regulate an individual’s mental health status before and after the onset of emotions (25, 26). Neurologists have further corroborated the mode of action of different emotion regulation strategies through functional MRI, potential recognition, and more (27, 28). Emotion regulation strategies have been applied in neurological disorders treatment, time perception, and psychological support and interventions (29–31). Studies have shown that cognitive reappraisal is beneficial in relieving emotions such as anxiety and depression, while expression suppression has the opposite effect (32–34). Consequently, we proposed the following two hypotheses:

*Hypothesis 2*: Cognitive reappraisal would negatively predict medical school students’ psychological responses.

*Hypothesis 3*: Expression suppression would positively predict medical school students’ psychological responses.

Emotional regulation strategies act as an interactive process between medical school students, school, and the development of the pandemic. They are not only strategies that medical school students have acquired during adolescence, but also post-educational and managerial processes developed during university study (26). Thus, emotional regulation strategies acquired and developed during the pandemic may be important for the future emotional management of students’ life and work. Different methods of campus closure management may lead to different emotional regulation strategies, which, in turn, may lead to different psychological experiences. Therefore, emotional regulation strategies may play a mediating role between campus lockdown and mental health, considering the sequential order of the two strategies (i.e., cognitive reappraisal and expression suppression) and their intrinsic connection with one another. Consequently, we proposed the following hypothesis:

*Hypothesis 4*: Emotional regulation strategies could play a mediating role between campus living conditions and mental health.

**Figure 1:**
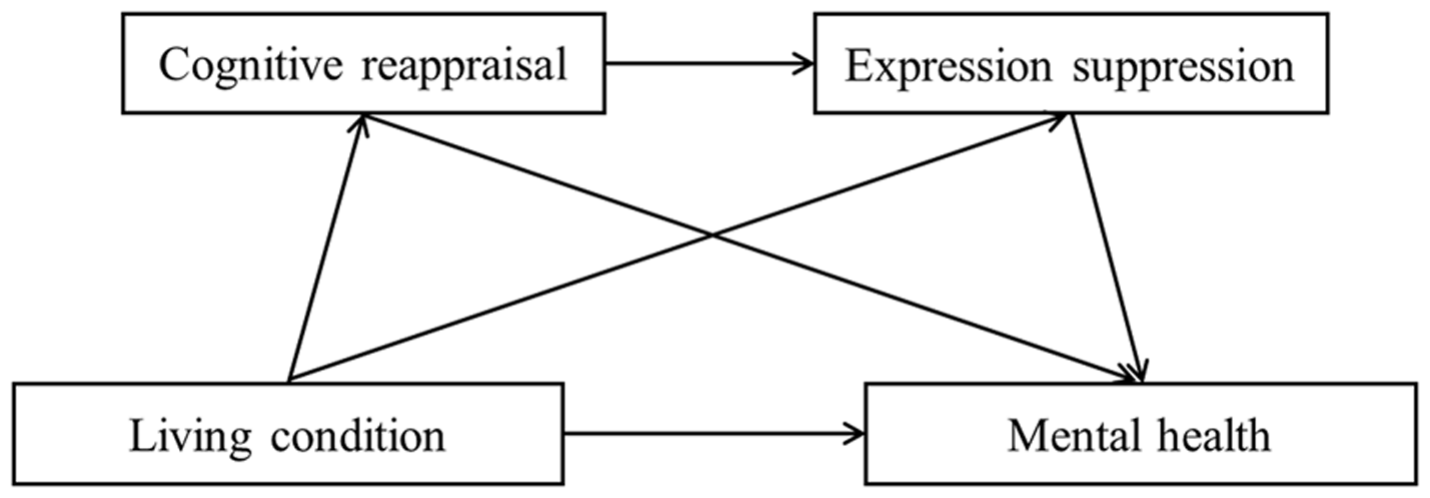
Pathway diagram of the chain mediating effect of “living condition – mental health.”

Therefore, this study analyzed the main factors and modes of action affecting medical school students’ mental health based on an investigation of their living conditions, emotion regulation strategies, and psychological reactions during campus lockdowns. This research can provide data to support universities in strengthening students’ mental health education, improving management services, and optimizing the path of medical talent training during the pandemic. Considering that cognitive reappraisal and expression suppression modulate an individual’s mental health before and after such emotions occur (25), the hypothetical model of the relationship between related variables is shown in Figure 1.

## 2. Methods

### 2.1. Participants and design

An anonymous survey was conducted from April to May 2022, which was the period of the campus lockdown included in this study during the COVID-19 pandemic. Students at a medical school were recruited from 11 departments *via* stratified random sampling. Thus, 10% (998) of the students on campus (about 10,000 students in total) were randomly selected according to grade stratification. Before the survey, all participants were briefed on the purpose, process, and privacy of the study by their tutors through online video conference and were required to answer the questions *via* the online questionnaire to facilitate data statistics and ensure that they meet the pandemic prevention requirements. The inclusion criteria were medical school students who experienced a campus lockdown for 1 week or longer and completed 80% or more of the questionnaire. A total of 998 college students were surveyed, and questionnaires that did not meet the internal consistency check requirements (*n* = 24) were excluded. Ultimately, 974 valid questionnaires were returned (total effective rate: 97.6%), of which 373 (38.3%) participants were men and 601 (61.7%) were women; 423 (43.4%) participants were 5-year medical majors, such as clinical medicine, dentistry, anesthesiology, forensic medicine, medical imaging, which means they will receive a medical degree after 5 years of study; and 551 (56.6%) were 4-year medical-related majors, such as nursing, pharmacy, medical testing, medical information, medical law and public health management, which means they have taken medical courses and will mostly work in hospitals or health facilities after graduation. The surveyed students were from one medical college of Anhui Province with an average age of 20.07 ± 1.76 years. After completing the questionnaire, the students were compensated with rewards or equivalent gifts for participating in the study. This study was performed in accordance with the ethical principles of the Declaration of Helsinki. The Medical Ethics Committee of Wannan Medical College approved this study and the format of the participant consent form (Approved No. 2022-102).

### 2.2. Measures

#### 2.2.1. Self-report questionnaire

A self-report questionnaire was used to collect participants’ basic demographic data and life situation information during the campus lockdown, including objective information such as “length of time under campus lockdown” and whether they “served as a volunteer supporting nucleic acid amplification tests,” and information on subjective experiences, such as “intensity of self-prevention,” “degree of inconvenience,” and “degree of change in routine.” The intensity of self-prevention was calculated based on the prevention and control measures noted in sources such as the “Prevention and Control Plan for Novel Coronavirus Pneumonia (8th Edition)” (35) and “Citizens’ Knowledge Manual for New Coronavirus Prevention” Version 2.0 (e.g., not leaving the dormitory or home, daily temperature measurement and health monitoring, nucleic acid amplification testing at least three times a week, daily window ventilation or disinfection, individual dining or no dine-in, and online work or study). Each effect was attributed one point, and total scores ranged from 0 to 8 points (“0” indicates “the least self-prevention,” and “8” signifies “the strictest self-prevention”). The degree of inconvenience in daily life was calculated based on Xie et al. (36) and included seven points (e.g., inconvenience in transportation, difficulty in accessing medical care, discomfort of being away from the crowd, and other aspects that require additional explanation). Each effect was attributed one point; thus, the total score ranged from 0 = “as convenient as before” to 7 = “extremely inconvenient.” For the degree of change in routine, participants used a scale ranging from 1 to 7 (from “not affected at all” to “completely affected”) to rate the degree to which their daily schedule was affected by the campus lockdown. This survey’s internal consistency coefficient was 0.609.

#### 2.2.2. Emotion regulation questionnaire

The Emotion Regulation Questionnaire (ERQ) (37) comprises 10 items and two dimensions: *cognitive reappraisal* and *expression suppression*. The participants rated their responses regarding situational emotion regulation strategies on a scale ranging from 1 = “completely disagree” to 7 = “completely agree.” The higher the mean score on the dimensions, the more participants were accustomed to using the emotion regulation strategy. In this study, the internal consistency coefficients for cognitive reappraisal and expression suppression were 0.934 and 0.852, respectively.

#### 2.2.3. Psychological questionnaires for emergent events of public health

The Psychological Questionnaires for Emergent Events of Public Health (PQEEPH) was developed by Chinese experts after the SARS pandemic in 2003. In addition, it was confirmed as applicable to the COVID-19 pandemic (38). The premise was set as “possible psychological reactions to the COVID-19 pandemic” to fit the actual situation of the study. The questionnaire comprises 25 items with five dimensions: *depression, neurosis, fear, obsessive–compulsive anxiety*, and *hypochondriasis*. The participants rated their responses to situational psychological reactions on a scale from 0 = “never” to 3 = “always.” Higher scores were associated with stronger psychological reactions. In this study, the internal consistency coefficients of the five dimensions were 0.928, 0.890, 0.792, 0.891, and 0.774, respectively.

### 2.3. Statistical analysis

SPSS Version 22.0 (IBM Corp., Armonk, NY, United States) was used to perform statistical analysis. In addition, the measurement data were expressed as “*x ± s*,” and the Wilcoxon rank sum test was used to compare differences between two groups (test value *Z*), the Kruskal–Wallis test was used to examine the differences between multiple groups (test value*χ2*), Spearman’s correlation analysis was used for each scale item and psychological response, and multiple linear stepwise regression analysis was performed for those with significance. The bootstrap method proposed by Preacher and Hayes (39) and Model 6 in the PROCESS macro program written by Hayes^1^ (40) were used to test the specific chain mediating effect. A test level of *α* = 0.05 and *p* < 0.05 was considered statistically significant.

## 3. Results

### 3.1. Common method bias

Harman’s single-factor test (41) was used to avoid common method bias in the self-report questionnaire, and the first common factor explained 36.87% of the variance. This result was less than the critical value of 40%, indicating that common method bias was not a serious issue in this study.

### 3.2. Descriptive statistics

#### 3.2.1. Descriptive statistics for living condition and emotion regulation strategies

Of the survey respondents, 658 (67.6%) reported being under campus lockdown management for up to 5–8 weeks, and 209 (21.5%) served as volunteers during campus lockdowns. Univariate analysis showed statistically significant differences in “intensity of self-prevention” based on whether participants were student cadres and volunteers (*Z* = −3.247, −2.646, *ps* < 0.05), “degree of inconvenience” based on year at college and whether they were student cadres (*Z/χ^2^* = 26.18, −3.289, *ps* < 0.05), and “degree of change in routine” based on gender (*Z* = −2.831, *p <* 0.05). Cognitive reappraisal scores differed statistically based on gender, major, whether they were student cadres, and whether they were volunteers (*Z* = −5.553, −2.326, −2.568, −2.305, *ps <* 0.05). No significant differences were found in expression suppression based on respondents’ general characteristics (Table 1).

**Table 1:** Medical school students’ living conditions, emotion regulation strategy scores under campus lockdowns (*N* = 974).

Features		*N*	Living situation			Emotional regulation strategies	
			Intensity of self-prevention	Degree of inconvenience	Degree of change in routine	Cognitive reappraisal	Expression suppression
Gender	Male	373	3.52 ± 1.6	2.52 ± 1.59	4.06 ± 1.81	4.21 ± 1.28	3.89 ± 1.26
	Female	601	3.64 ± 1.44	2.52 ± 1.51	3.73 ± 1.72	4.64 ± 1.07	3.84 ± 1.15
	*Z*		−1.11	−0.406	−2.831**	−5.553***	−1.467
Major	Medical	423	3.6 ± 1.45	2.56 ± 1.56	3.84 ± 1.8	4.57 ± 1.16	3.85 ± 1.24
	Medical related	551	3.59 ± 1.54	2.48 ± 1.53	3.87 ± 1.73	4.4 ± 1.18	3.86 ± 1.16
	*Z*		−0.212	−0.75	−0.463	−2.326*	−0.301
Year at college	1st	377	3.58 ± 1.56	2.29 ± 1.44	3.77 ± 1.64	4.42 ± 1.25	3.87 ± 1.21
	2nd	289	3.71 ± 1.44	2.39 ± 1.38	3.89 ± 1.64	4.56 ± 1.14	3.85 ± 1.26
	3rd	151	3.54 ± 1.32	2.88 ± 1.77	3.79 ± 1.93	4.37 ± 0.93	3.95 ± 0.95
	4th	103	3.53 ± 1.51	3.04 ± 1.7	3.88 ± 2.06	4.55 ± 1.3	3.77 ± 1.27
	5th	54	3.35 ± 1.82	2.8 ± 1.71	4.41 ± 2	4.54 ± 1.21	3.72 ± 1.28
	*χ^2^*		4.31	26.18***	5.521	7.673	1.116
Student cadre participation	Yes	302	3.81 ± 1.48	2.74 ± 1.57	3.95 ± 1.74	4.61 ± 1.2	3.89 ± 1.21
	No	672	3.49 ± 1.50	2.42 ± 1.52	3.81 ± 1.77	4.41 ± 1.16	3.84 ± 1.19
	*Z*		−3.247**	−3.289**	−1.041	−2.568*	−0.397
Volunteer status	Yes	209	3.83 ± 1.63	2.51 ± 1.48	3.82 ± 1.73	4.63 ± 1.27	3.95 ± 1.25
	No	765	3.53 ± 1.46	2.52 ± 1.56	3.87 ± 1.77	4.43 ± 1.14	3.83 ± 1.18
	*Z*		−2.646**	−0.413	−0.227	−2.305*	−1.06
Lockdown duration	1–2 weeks	19	3.37 ± 2.03	2.21 ± 1.81	4.00 ± 2.00	4.34 ± 0.98	4.12 ± 1.11
	3–4 weeks	52	3.58 ± 1.39	2 ± 0.97	3.87 ± 1.70	4.34 ± 1.39	3.76 ± 1.34
	5–8 weeks	658	3.57 ± 1.51	2.5 ± 1.54	3.79 ± 1.71	4.51 ± 1.13	3.83 ± 1.15
	9–12 weeks	205	3.69 ± 1.47	2.65 ± 1.59	4.01 ± 1.83	4.45 ± 1.23	3.91 ± 1.23
	12 weeks or more	40	3.55 ± 1.47	2.9 ± 1.77	4.1 ± 2.19	4.24 ± 1.43	4.09 ± 1.52
	*χ^2^*		1.445	9.448	2.659	1.908	3.222

Medical-related fourth-year students were counted as fifth-year students; *p < 0.05, **p < 0.01, ***p < 0.001.

#### 3.2.2. Descriptive statistics for mental health

The mean total PQEEPH score during the campus lockdowns was 3.66 ± 3.06, and the mean scores of each dimension from highest to lowest were fear (1 ± 0.62), depression (0.89 ± 0.8), neurosis (0.84 ± 0.74), obsessive–compulsive-anxiety (0.48 ± 0.64), and hypochondriasis (0.45 ± 0.64). In addition, univariate analysis showed statistically significant differences in depression scores based on lockdown duration (*χ^2^* = 11.4, *p* < 0.05); neurosis scores based on major, year at college, volunteer status, and lockdown duration (*Z/χ^2^* = −2.612, 19.771, −2.636, 11.938, *ps* < 0.05); fear scores based on gender, year at college, and lockdown duration (*Z/χ^2^* = −2.866, 10.922, 13.133, *ps* < 0.05); obsessive–compulsive-anxiety scores based on year at college and lockdown duration (*χ^2^* = 13.034, 17.231, *ps* < 0.05); and hypochondriasis scores based on lockdown duration (*χ^2^* = 21.133, *p* < 0.05; Table 2).

**Table 2:** Mental health dimension scores of medical school students (*N* = 974).

Features		Depression	Neurosis	Fear	Obsessive–compulsive-anxiety	Hypochondriasis
Gender	Male	0.91 ± 0.82	0.81 ± 0.77	0.94 ± 0.66	0.53 ± 0.71	0.51 ± 0.71
	Female	0.88 ± 0.78	0.86 ± 0.71	1.03 ± 0.59	0.44 ± 0.59	0.42 ± 0.59
	*Z*	−0.342	−1.712	−2.866**	−0.703	−1.223
Major	Medical	0.91 ± 0.83	0.91 ± 0.78	1.03 ± 0.64	0.51 ± 0.70	0.49 ± 0.70
	Medical related	0.88 ± 0.78	0.78 ± 0.7	0.96 ± 0.6	0.45 ± 0.58	0.42 ± 0.59
	*Z*	−0.119	−2.612**	−1.281	−0.567	−0.984
Year at college	1st Year	0.85 ± 0.74	0.71 ± 0.65	0.92 ± 0.56	0.39 ± 0.56	0.39 ± 0.57
	2nd Year	0.89 ± 0.76	0.84 ± 0.72	1.01 ± 0.61	0.47 ± 0.61	0.46 ± 0.63
	3rd Year	0.99 ± 0.88	1.05 ± 0.87	1.13 ± 0.66	0.64 ± 0.72	0.58 ± 0.73
	4th Year	0.93 ± 0.90	0.93 ± 0.76	0.96 ± 0.71	0.50 ± 0.72	0.46 ± 0.71
	5th Year	0.89 ± 0.96	0.95 ± 0.86	1.06 ± 0.74	0.55 ± 0.80	0.52 ± 0.76
	*χ^2^*	2.272	19.771**	10.922*	13.034*	7.544
Student cadre participation	Yes	0.92 ± 0.81	0.82 ± 0.73	1.00 ± 0.63	0.48 ± 0.65	0.45 ± 0.64
	No	0.88 ± 0.79	0.85 ± 0.74	0.99 ± 0.62	0.48 ± 0.63	0.45 ± 0.64
	*Z*	−0.796	−0.591	−0.539	−0.082	−0.048
Volunteer status	Yes	0.85 ± 0.84	0.74 ± 0.75	0.93 ± 0.65	0.46 ± 0.69	0.44 ± 0.71
	No	0.91 ± 0.79	0.86 ± 0.73	1.01 ± 0.61	0.48 ± 0.63	0.45 ± 0.62
	*Z*	−1.499	−2.636**	−1.789	−1.297	−1.257
Lockdown duration	1–2 weeks	1.14 ± 0.82	1.25 ± 0.84	1.3 ± 0.7	0.92 ± 0.69	1.08 ± 0.84
	3–4 weeks	0.81 ± 0.80	0.83 ± 0.74	0.95 ± 0.63	0.50 ± 0.65	0.46 ± 0.70
	5–8 weeks	0.84 ± 0.75	0.80 ± 0.69	0.95 ± 0.58	0.42 ± 0.56	0.40 ± 0.56
	9–12 weeks	0.99 ± 0.87	0.85 ± 0.81	1.05 ± 0.68	0.54 ± 0.75	0.49 ± 0.71
	12 weeks or more	1.27 ± 1.05	1.21 ± 0.98	1.30 ± 0.83	0.83 ± 0.97	0.80 ± 0.99
	*χ^2^*	11.4*	11.938*	13.133*	17.231***	21.133***

*p < 0.05, **p < 0.01, ***p < 0.001.

### 3.3. Factors influencing mental health during campus lockdown management

Spearman’s correlation analysis showed that the degree of inconvenience and change in routine, cognitive reappraisal, expression suppression, and the five PQEEPH dimensions were significantly correlated (*r* = −0.114 ~ 0.387, *ps* < 0.05), except for cognitive reappraisal, which was not significantly associated with fear. The intensity of self-prevention was only significantly correlated with fear (*r* = 0.096, *p* < 0.01).

The scores of the five PQEEPH dimensions were used as dependent variables. The variables that were meaningful for each dimension of univariate analysis, such as gender, major, year at college, volunteer status (coded in the same way as 2.2), lockdown duration (1–2 weeks, 3–4 weeks, 5–8 weeks, 9–12 weeks, and more than 12 weeks were coded as 0–4, respectively), and the scores of each scale were used as independent variables for further multiple linear stepwise regression analysis. The results showed that depression (*R^2^* = 0.326, *F* = 93.664, *p* < 0.001), neurosis (*R^2^* = 0.291, *F* = 49.431, *p* < 0.001), fear (*R^2^* = 0.184, *F* = 31.106, *p* < 0.001), obsessive–compulsive-anxiety (*R^2^* = 0.27, *F* = 44.583, *p* < 0.001), and hypochondriasis (*R^2^* = 0.164, *F =* 23.621, *p* < 0.001) were significant for all five-dimensional regression model effects. Consistent with Hypotheses 1, 2, and 3, degree of change in routine, degree of inconvenience, and expression suppression were significant positive predictors of all five psychological response dimensions, and cognitive reappraisal was a significant negative predictor of four dimensions, excluding fear. Being under campus lockdown for 3–12 weeks was a significant negative predictor of obsessive–compulsive anxiety and hypochondriasis, 5–8 weeks was a significant negative predictor of depression and fear, with 12 weeks or more being a significant positive predictor of neurosis, fear, and a significant negative predictor of hypochondriasis. Being in the third year of study significantly positively predicted neurosis, fear, and obsessive–compulsive anxiety. Neurosis was significantly positively predicted by having a medical-related major and significantly negatively predicted by not being a volunteer. Female gender significantly positively predicted fear (Table 3).

**Table 3:** Regression model of factors influencing medical school students’ mental health.

Independent variable	Depression		Neurosis		Fear		Obsessive–compulsive anxiety		Hypochondriasis	
	*Beta*	*t*	*Beta*	*t*	*Beta*	*t*	*Beta*	*t*	*Beta*	*t*
Degree of change in routine	0.353	11.888***	0.295	9.666***	0.241	7.369***	0.253	8.167***	0.196	5.916***
Degree of inconvenience	0.243	8.232***	0.216	7.057***	0.16	4.896***	0.202	6.5***	0.123	3.705***
Expression suppression	0.21	7.011***	0.225	7.28***	0.177	6.061***	0.238	7.599***	0.208	6.204***
Cognitive reappraisal	−0.099	−3.283**	−0.123	−3.929***	/	/	−0.198	−6.275***	−0.158	−4.679***
Lockdown duration										
3–4 weeks	/	/	/	/	/	/	−0.084	−2.29*	−0.196	−3.537***
5–8 weeks	−0.063	−2.393*	/	/	−0.065	−2.125*	−0.247	−4.505***	−0.461	−4.598***
9–12 weeks	/	/	/	/	/	/	−0.174	−3.36**	−0.371	−4.126***
12 weeks or more	/	/	0.073	2.681**	0.061	1.99*	/	/	−0.102	−1.995*
3rd year	/	/	0.098	3.559***	0.08	2.713**	0.08	2.877**	/	/
Medical related	/	/	−0.097	−3.531***	/	/	/	/	/	/
Non-volunteer	/	/	0.06	2.214*	/	/	/	/	/	/
Female	/	/	/	/	0.105	3.574***	/	/	/	/

*p < 0.05, **p < 0.01, ***p < 0.00; univariate analysis differences were not statistically significant, and linear regression *p* ≥ 0.05 data have been removed.

### 3.4. Testing of the mediated model of medical school students’ mental health

To further explore the role of emotion regulation strategies in the relationship between medical school students’ campus living conditions and mental health, the survey data were analyzed and processed according to the mediating effect test procedure proposed by Wen and Ye (42). The degrees of change in routine and inconvenience were taken as independent variables X_1_ and X_2_, respectively. Cognitive reappraisal was mediating variable M_1_, and expression suppression was mediating variable M_2_. The total PQEEPH score was dependent variable Y. Model 6 was used to perform mediation analysis, with lockdown duration, year at college, major, volunteer status, and gender as covariates, which were standardized for each variable (Table 4).

**Table 4:** Regression analysis of variables in the intermediary model.

Regression equation		Overall fit index			Significance of regression coefficients		95% CI
Result variables	Predictive variables	*R*	*R^2^*	*F*	*B*	*t*	
X_1_ = Degree of inconvenience							
Cognitive reappraisal	Degree of inconvenience	0.226	0.051	8.654	−0.062	−2.562*	[−0.11, −0.015]
Expression suppression	Degree of inconvenience	0.485	0.235	42.397	0.078	3.514***	[0.035, 0.122]
	Cognitive reappraisal				0.499	16.985***	[0.442, 0.557]
Mental health	Degree of inconvenience	0.463	0.214	32.883	0.683	11.728***	[0.569, 0.798]
	Cognitive reappraisal				−0.503	−5.783***	[−0.673, −0.332]
	Expression suppression				0.711	8.515***	[0.547, 0.875]
X_2_ = Degree of change in routine							
Cognitive reappraisal	Degree of change in routine	0.239	0.057	9.8	−0.076	−2.562***	[−0.117, −0.035]
Expression suppression	Degree of change in routine	0.482	0.233	41.837	0.059	3.0516**	[0.021, 0.097]
	Cognitive reappraisal				0.501	16.967***	[0.443, 0.559]
Mental health	Degree of change in routine	0.504	0.254	41.051	0.69	14.008***	[0.594, 0.787]
	Cognitive reappraisal				−0.455	−5.35***	[−0.673, −0.288]
	Expression suppression				0.71	8.738***	[0.547, 0.869]

*p < 0.05, **p < 0.01, ***p < 0.00.

Significance analysis of the mediating effects was performed using the bias-corrected percentile bootstrap method (repeated sampling 5,000 times, 95% confidence interval [CI]). The results showed that after controlling for the mediating variables (cognitive reappraisal and expression suppression), the direct effect of the degree of inconvenience on the dependent variable (mental health) was significant, with an interval that did not contain 0 (effect = 0.683, 95% CI: [0.569, 0.798]). In addition, the direct impact of the degree of change in routine on the dependent variable (mental health) was also significant, with an interval that did not contain 0 (effect = 0.69, SE = 0.493, 95% CI: [0.594, 0.787]).

**Figure 2:**
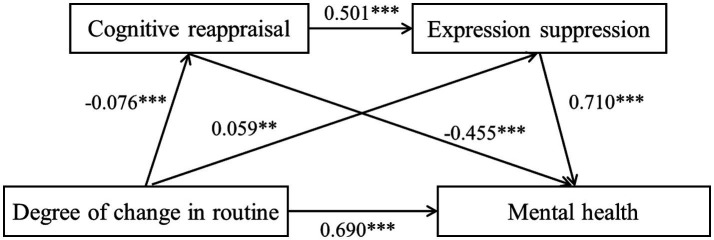
Pathway diagram of the chain mediating effect of “degree of inconvenience – mental health.”

Consistent with Hypothesis 4, the chain mediating effects of cognitive reappraisal and expression suppression were all significant. Degree of inconvenience significantly affected mental health through three paths: ① degree of inconvenience → cognitive reappraisal → mental health (effect = 0.031, SE = 0.018, 95% CI: [0.006, 0.064]), ② degree of inconvenience → expression suppression → mental health (effect = 0.056, SE = 0.018, 95% CI: [0.023, 0.092]), and ③ degree of inconvenience → cognitive reappraisal → expression suppression → mental health (effect = −0.022, SE = 0.01, 95% CI: [−0.042, −0.005]). The mediating effects of paths ① and ② were significant and accounted for 12.73% of the direct effect. The masking effect of path ③ was significant and accounted for 3.23% of the direct effect. The results are shown in Table 5, and the specific paths are shown in Figure 2.

**Table 5:** Significance analysis of the mediation model.

Impact path	95% CI	Effect value	Bootstrap standard error	Indirect effects as a proportion of direct effects
X_1_ = Degree of inconvenience				
Total indirect effect	[0.03, 0.103]	0.065	0.018	9.51%
① Degree of inconvenience → Cognitive reappraisal → Mental health	[0.006, 0.064]	0.031	0.015	4.58%
② Degree of inconvenience → Expression suppression → Mental health	[0.023, 0.092]	0.056	0.018	8.15%
③ Degree of inconvenience → Cognitive reappraisal → Expression suppression → Mental health	[−0.042, −0.005]	−0.022	0.01	3.23%
Sum of intermediary effects	① + ②	0.087		12.73%
Sum of masking effects	③	−0.022		3.23%
X_2_ = Degree of change in routine				
Total indirect effect	[0.019, 0.084]	0.05	0.017	7.18%
④ Degree of change in routine→ Cognitive reappraisal→ Mental health	[0.011, 0.064]	0.035	0.013	5.01%
⑤ Degree of change in routine → Expression suppression → Mental health	[0.013, 0.075]	0.042	0.016	6.1%
⑥ Degree of change in routine → Cognitive reappraisal → Expression suppression → Mental health	[−0.046, −0.01]	−0.027	0.009	3.93%
Sum of intermediary effects	④ + ⑤	0.077		11.11%
Sum of masking effects	⑥	−0.027		3.93%

**Figure 3:**
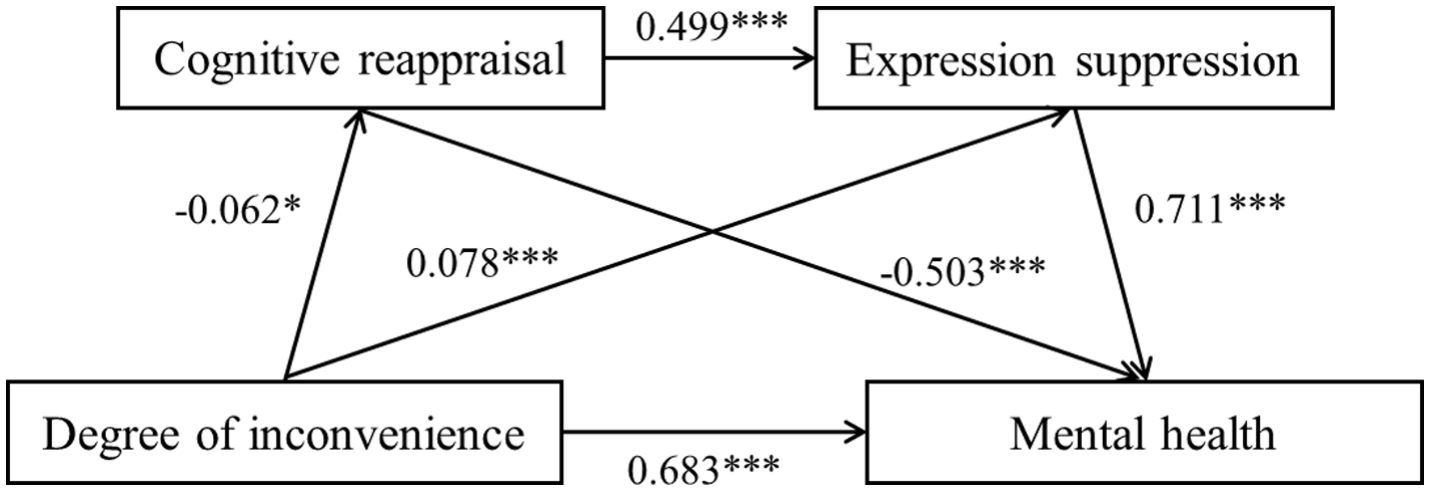
Pathway diagram of the chain mediating effect of “degree of change in routine – mental health.”

The degree of change in routine also significantly affected mental health through three paths: ④ degree of change in routine → cognitive reappraisal → mental health (effect = 0.035, SE = 0.015, 95% CI: [0.011, 0.064]), ⑤ degree of change in routine → expression suppression → mental health (effect = 0.042, SE = 0.018, 95% CI: [0.013, 0.075]), and ⑥ degree of change in routine → cognitive reappraisal → expression suppression → mental health (effect = −0.027, SE = 0.01, 95% CI: [−0.046, −0.01]). The mediating effect of paths ④ and ⑤ was significant and accounted for 11.11% of the direct effect. The masking effect of path ⑥ was significant and accounted for 3.93% of the direct effect. The results are shown in Table 5, and the specific paths are shown in Figure 3.

## 4. Discussion

This study examined the role of campus lockdown management on medical school students’ mental health during the COVID-19 pandemic. The results showed that living conditions could directly affect mental health, and cognitive reappraisal and expression suppression played partial mediating and partial chain mediating roles in that relationship.

### 4.1. Objective factors affecting medical school students’ mental health

Campus lockdown duration, year at college, major, volunteer status, gender, and other objective factors have a specific impact on mental health. Campus lockdowns for “1–2 weeks” and “3 months and more” led to a high incidence of psychological reactions in students. Third-year students reported more neurosis, fear, and obsessive–compulsive anxiety than other students, while female students had a stronger fear reaction than male students. An additional explanation of the self-report questionnaire showed that the main reasons for students’ psychological reactions during the “1–2 weeks” lockdown were changes in the rhythm of campus life and fear of the virus, which gradually subsided after 2 weeks. With the continuous extension of lockdowns, students could not foresee a return to normal life, which exacerbated their adverse psychological responses.

Although the third-year students had started their clinical internships, the COVID-19 pandemic campus lockdown interrupted them. Thus, the students felt that the pandemic strongly affected their studies, which is consistent with the findings of Wang et al. (43). The study also showed that female students had a more pronounced fear response than males, which is consistent with the findings of Xu and Huang (44) and Metin et al. (45) – fear can lead to worse mental health outcomes: female students were more likely to show “easy-to-acquire, hard-to-abate” characteristics concerning memories of fear, owing to their physiology and estrogen levels. This finding suggests that more attention needs to be paid to students’ psychological reactions during the initial period of campus lockdowns and for lockdowns that last 3 months or more. Therefore, colleges should implement measures, such as helping students resolve their worries, relieve stress, overcome fears, and improve their coping efficacy related to the pandemic, through mental health education and knowledge dissemination on pandemic prevention and control (46). Furthermore, colleges should also organize students to participate in campus pandemic prevention and control propaganda and volunteer services, organize non-group outdoor activities to enrich students’ campus lives, and offer services such as distance learning, virtual simulation teaching, and clinical skills training to reduce the pandemic’s impact on third-year students’ internships.

### 4.2. Living conditions affecting medical school students’ mental health

Although previous studies (12–16) have focused on objective factors, such as lockdown duration and location, they have neglected to examine subjective factors. To address this gap, this study measured subjective factors related to lockdowns. The preliminary results showed that subjective characteristics, such as perceived changes in routine and inconvenience in daily life, significantly affected mental health during lockdowns, compared with objective factors such as lockdown duration and volunteer status. This suggests that colleges should focus on students who have been under extended lockdown and non-volunteers to identify people with poor mental health and should also consider students’ personal feelings during the campus lockdown process. Furthermore, this strategy would contribute to identifying and responding effectively to students’ psychological problems during campus lockdown management.

Students’ subjective perceptions of daily life inconveniences and routine changes during campus lockdowns were closely related to their mental health. The higher the degree of inconvenience and change in daily routine, the higher the scores on the five psychological reaction dimensions and the poorer the mental health outcomes. During campus lockdowns, students are restricted to the campus. Changes in their routines, a narrowed scope of life and study activities, and inconveniences in daily life increase the possibility of students developing psychological problems. Various types of support and recreational facilities should be provided during campus lockdown to enrich campus life and provide students with the necessary psychological support. This could guide students toward adopting positive emotional regulation strategies and channels for discussing worries and relieving negative emotions, thereby providing them with appropriate psychoeducation on controlling their frustration. These prudent measures could effectively promote the mental health development of medical school students.

### 4.3. Mediating role of emotion regulation between living conditions and mental health

Cognitive reappraisal modulates emotions before they occur and alters emotional reactions, experiences, and unpleasant moods, significantly reducing the risk of emotional distress and other mental health problems. Conversely, expression suppression strategies targeting emotions, such as depression, obsessions, and neurosis, within an individual worsen emotional problems. When the degree of inconvenience was the independent variable, it significantly negatively predicted cognitive reappraisal and significantly positively predicted expression suppression. Furthermore, cognitive reappraisal significantly positively predicted expression suppression. When the degrees of inconvenience, cognitive reappraisal, and expression suppression were entered into the regression equation simultaneously, they all positively predicted mental health, indicating that cognitive reappraisal and expression suppression partially mediated the degree of inconvenience and mental health, respectively.

By contrast, cognitive reappraisal and expression suppression mediated the chain effect between them. The same was true when the independent variable was the degree of change in routine. This result shows that cognitive reappraisal and emotion regulation strategies (particularly those involving expression suppression) mediate between campus lockdown living conditions and mental health. Subjective experiences, such as inconvenience in daily life and changes in routine related to campus lockdowns, may reduce the use of cognitive reappraisal, thereby threatening mental health. However, feelings of helplessness regarding the current situation may further increase the use of expression suppression and intensify psychological reactions. Thus, cognitive reappraisal is conducive to reducing adverse psychological reactions, while expression suppression elicits the opposite. This finding is generally consistent with Xu et al. (47) and Akbari et al. (48) regarding the mediating role of different emotion regulation strategies between external stimuli and psychological reactions.

Notably, using cognitive reappraisal before the onset of emotions and expression suppression during the onset of emotions can reduce psychological reactions to some extent in the context of campus lockdowns; this complements Gross’s classical emotion regulation strategy theory (24, 25) on the roles of both strategies. This study’s findings show that the integrated use of both emotion regulation strategies is an effective method for improving mental health during campus lockdown management, which provides new ideas for active prevention of, and intervention against college students’ mental health problems.

## 5. Limitations and future research

This study was based on data obtained during the most stringent campus lockdown period of the pandemic. With the introduction of new policies to control the pandemic in China, this strict closure environment may be challenging to recreate. In addition, this study was mainly based on quantitative research and lacks qualitative research, although the subjective and objective factors of campus lockdown were considered as much as possible. In-depth interviews with relevant stakeholders, such as administrators, teachers, and students, should be more prominent in future studies. Finally, the context of Anhui Province is very different from other provinces, regions, and countries. Further comparison of data from different contexts to identify similarities and differences is necessary for future studies.

## 6. Conclusion

The results of this study show that emotion regulation is a potential mediating mechanism in the relationship between the degree of inconvenience, the degree of change in routine, and mental health. In addition, cognitive reappraisal and expression suppression have a partial mediation effect between the degree of inconvenience and mental health and the degree of change in routine and mental health.

## Data availability statement

The raw data supporting the conclusions of this article will be made available by the authors, without undue reservation.

## Ethics statement

The studies involving human participants were reviewed and approved by the Medical Ethics Committee of Wannan Medical College. The patients/participants provided their written informed consent to participate in this study.

## Author contributions

WZ contributed to the conception and design of the study, and wrote the first draft of manuscript. LH and FX wrote sections of the manuscript. All authors contributed to the manuscript revision and have read and approved the submitted version.

## Funding

This study was supported by the Major Project of Humanities and Social Sciences of the Anhui Provincial Education Department (Grant No. SK2019ZD19), the National College Students’ Innovation and Entrepreneurship Training Program of China (Grant No. 202210368026) and the Special Project on Ideological and Political Education for Counselors of Wannan Medical College (Grant No. SZ201902).

## Conflict of interest

The authors declare that the research was conducted in the absence of any commercial or financial relationships that could be construed as a potential conflict of interest.

## Publisher’s note

All claims expressed in this article are solely those of the authors and do not necessarily represent those of their affiliated organizations, or those of the publisher, the editors and the reviewers. Any product that may be evaluated in this article, or claim that may be made by its manufacturer, is not guaranteed or endorsed by the publisher.

## Footnotes

^1^ https://www.processmacro.org/download.html
