# Supplementary material for: Impact of campus living conditions on Chinese medical school students’ mental health during the COVID-19 campus lockdown: the chain mediating role of cognitive reappraisal and expression suppression
Source: Front Psychiatry. 2023 May 17;14:1171425. doi: 10.3389/fpsyt.2023.1171425 (PMC10229781; doi:10.3389/fpsyt.2023.1171425)
Supplement: Supplementary file 1 [file Table_1.DOCX]

**Annexes: Table 3 Raw data before merging**

| **Regression model of factors influencing medical students’ Depression** | | | |
| --- | --- | --- | --- |
|  | *Beta* | *t* | *p* |
| Constants |  | -1.871 | 0.062 |
| Degree of change in routine | 0.353 | 11.888 | 0 |
| Degree of inconvenience | 0.243 | 8.232 | 0 |
| Expression suppression | 0.21 | 7.011 | 0 |
| Cognitive reappraisal | -0.099 | -3.283 | 0.001 |
| Lockdown duration 5-8weeks | -0.063 | -2.393 | 0.017 |
|  |  |  |  |
| **Regression model of factors influencing medical students’ Neurosis** | | | |
|  | *Beta* | *t* | *p* |
| Constants |  | -1.18 | 0.238 |
| Degree of change in routine | 0.295 | 9.666 | 0 |
| Degree of inconvenience | 0.216 | 7.057 | 0 |
| Expression suppression | 0.225 | 7.28 | 0 |
| Cognitive reappraisal | -0.123 | -3.929 | 0 |
| Medical related | -0.097 | -3.531 | 0 |
| 3rd year | 0.098 | 3.559 | 0 |
| Lockdown 12 weeks or more | 0.073 | 2.681 | 0.007 |
| Non-Volunteer | 0.06 | 2.214 | 0.027 |
|  |  |  |  |
| **Regression model of factors influencing medical students’ Fear** | | | |
|  | *Beta* | *t* | *p* |
| Constants |  | 1.157 | 0.248 |
| Degree of change in routine | 0.241 | 7.369 | 0 |
| Expression suppression | 0.177 | 6.061 | 0 |
| Degree of inconvenience | 0.16 | 4.896 | 0 |
| Female | 0.105 | 3.574 | 0 |
| Lockdown duration 5-8 weeks | -0.065 | -2.125 | 0.034 |
| 3rd year | 0.08 | 2.713 | 0.007 |
| Lockdown 12 weeks or more | 0.061 | 1.99 | 0.047 |

| **Regression model of factors influencing medical students’ Obsessive-compulsive anxiety** | | | |
| --- | --- | --- | --- |
|  | *Beta* | *t* | *p* |
| Constants |  | 1.57 | 0.117 |
| Degree of change in routine | 0.253 | 8.167 | 0 |
| Degree of inconvenience | 0.202 | 6.5 | 0 |
| Expression suppression | 0.238 | 7.599 | 0 |
| Cognitive reappraisal | -0.198 | -6.275 | 0 |
| Lockdown duration 5-8weeks | -0.247 | -4.505 | 0 |
| 3rd year | 0.08 | 2.877 | 0.004 |
| Lockdown duration 9-12weeks | -0.174 | -3.36 | 0.001 |
| Lockdown duration 3-4weeks | -0.084 | -2.29 | 0.022 |
|  |  |  |  |
| **Regression model of factors influencing medical students’ Hypochondriasis** | | | |
|  | *Beta* | *t* | *p* |
| Constants |  | 3.646 | 0 |
| Degree of change in routine | 0.196 | 5.916 | 0 |
| Expression suppression | 0.208 | 6.204 | 0 |
| Cognitive reappraisal | -0.158 | -4.679 | 0 |
| Degree of inconvenience | 0.123 | 3.705 | 0 |
| Lockdown duration 5-8weeks | -0.461 | -4.598 | 0 |
| Lockdown duration 9-12weeks | -0.371 | -4.126 | 0 |
| Lockdown duration 3-4weeks | -0.196 | -3.537 | 0 |
| Lockdown 12 weeks or more | -0.102 | -1.995 | 0.046 |
